# Spermatorrhœa—Impotence—Cure

**Published:** 1884-09-20

**Authors:** C. A. Bryce

**Affiliations:** Richmond, Va.


### SPERMATORRHŒA—IMPOTENCE—CURE.

By C. A. Bryce, M.D., Richmond, Va.

Some years ago the writer published an article in this journal under the caption of "Psychical Impotence" which was extensively copied into the journals of this country, as well as across the water in both the French and German languages. The object of that paper was to point out the reason why many cases of impotency were not cured, as well as to suggest the rational management of the same. I propose, in this paper, to review the history and successful treatment of a most distressing case, which I have recently restored to perfect health; and, as the patient had been under the treatment of several excellent physicians without receiving benefit, it may be of service to my professional friends if I succeed in pointing out the cause of failure on the part of my estimable friends who had been treating this patient before he fell into my hands. A report of this case in detail will be the best way to properly introduce it to my readers. Early in the present year I was consulted by a young gentleman, some twenty-five years of age, for a trouble or series of ailments which were described as follows:

"For two years I have had not a single erection of the organ of generation. I have no sexual feelings or desires. If, by chance, anything should somewhat feebly arouse my feelings, I have a seminal discharge without an erection. I have, also, a most frequent and urgent desire to urinate every hour of the day, and frequently have to arise at night to empty the bladder. Any excitement of any kind will cause me to void the urine, and if I cannot find a suitable place for the purpose it is passed in my pants."

This was certainly not a very encouraging case, and the young man had sought us through the advice of some patient who had been treated by us for some similar trouble. He was very much discouraged, and twice asked to be relieved from a marriage engagement which still existed. He desired to be cured, if such were possible, and, if not, to have me state frankly his condition, that he might break off an engagement which he felt would be wrong to consummate under the existing circumstances. We asked him what treatment had been pursued. He was au fait on the remedies, from beginning to end, and ran over: "Iron, strychnia, phosphorus, zinc, damiana, cold baths, counter irritation," etc., with remarkable familiarity. In answer to our query as to whether any surgeon who had been treating him had ever passed an instrument into his bladder, or made an *instrumental* examination of him, he replied with a very decided and prompt "No, sir, never, none of them!" It occurred to us as remarkably strange, how so important a measure could have been overlooked or neglected by his former attendants. We accordingly informed him that we would be under the necessity of making such an examination before we could do anything for him. He very nervously consented, and we made the attempt to pass a No. 18 steel sound into the bladder. The sound was not passed two inches within the urethra before he begged us to desist, saying that he could not possibly stand it, as it made him very giddy. The sound was firmly gripped, and showed that the man had urethral hyperæsthesia. He was given a little bromide of potassium to take, and ordered to return in three days. At his second visit he was similarly affected by the attempt to pass the instrument, but it was passed further down at this trial. To shorten the account, we will state that he made five visits to our office before we succeeded in introducing a full-sized sound clearly into the bladder. Not a particle of medicine was given this patient, but a No. 21 F. steel sound was passed into the bladder twice a week for five weeks. At the end of that time his condition was as follows:

Passes the urine three times a day—when the bladder is well filled; has no sudden or spasmodic desire to pass the urine, and can hold it until he can find it convenient to empty the bladder. Has had neither seminal emissions by day, nor nocturnal pollutions. Frequently during the week he has firm and natural erections without any emission of semen. Has recovered from his great nervousness and feels well and strong as ever, in every way. Has subsequently married and reports himself satisfied and giving eminent satisfaction to all parties concerned! With the exception of a little potass. bromid. and, subsequently, ferri dialyzatum, this man was cured absolutely *without medicine*. The most difficult portion of the treatment consisted in assuring the patient that he *could be cured*, and in allaying his fears as to his future sexual integrity. After we had properly educated him as to the physiology and pathology bearing on his case, he became a hopeful and obedient patient and responded in a most happy manner to everything that was done for his relief. The mental part of the treatment in such cases is all-important and should never be overlooked. To attach *too much importance*, however, to mental influence at the expense of medicinal or surgical treatment is equally as grave a mistake.—*Southern Clinic.*

